# On the Post-mortem Duration of the Ciliary Movements in the Human Subject

**Published:** 1851-11

**Authors:** 


*On the Post-mortem Duration of the Ciliary Movements in the Human Subject.* By M. GOSSELIN.—The body of a decapitated criminal having been conveyed to the Ecole Pratique, the ciliary movement was recognized on the mucous membrane of the trachea, of the nasal fossæ, and on that lining the maxillary, frontal, and sphenoidal sinuses, eight hours after death. The movements were still distinguishable, especially on the mucous membrane of the trachea, 32 hours after death. The movement had ceased on the mucous membrane of the nasal fossæ and of the sinuses, 56 hours after death; but this was perhaps due to the free exposure of these parts to the air; for the vibration was still active on the mucous membrane of the trachea, where it was distinctly seen to the 168th hour after death, after which putrefaction came on, and the movement ceased. In another case of the same nature, the ciliary movements were much less durable; and this seemed to be consequent upon the earlier supervention of putrefaction, brought about by a higher temperature, the thermometer having ranged from 46° to 54° in the first and having risen to 68° in the second.—*Ibid, from Gazette Médicale.*

